# Imaging Hallmarks of the Tumor Microenvironment in Glioblastoma Progression

**DOI:** 10.3389/fonc.2021.692650

**Published:** 2021-08-26

**Authors:** John J. Walsh, Maxime Parent, Adil Akif, Lucas C. Adam, Samuel Maritim, Sandeep K. Mishra, Muhammad H. Khan, Daniel Coman, Fahmeed Hyder

**Affiliations:** ^1^Department of Biomedical Engineering, Yale University, New Haven, CT, United States; ^2^Department of Radiology and Biomedical Imaging, Yale University, New Haven, CT, United States; ^3^Magnetic Resonance Research Center, Yale University, New Haven, CT, United States

**Keywords:** glioblastoma, vascularity, extracellular pH, tumor microenvironment, cellularity, chemical shift imaging, dynamic contrast enhanced imaging, MRI

## Abstract

Glioblastoma progression involves multifaceted changes in vascularity, cellularity, and metabolism. Capturing such complexities of the tumor niche, from the tumor core to the periphery, by magnetic resonance imaging (MRI) and spectroscopic imaging (MRSI) methods has translational impact. In human-derived glioblastoma models (U87, U251) we made simultaneous and longitudinal measurements of tumor perfusion (F_p_), permeability (K^trans^), and volume fractions of extracellular (v_e_) and blood (v_p_) spaces from dynamic contrast enhanced (DCE) MRI, cellularity from apparent diffusion coefficient (ADC) MRI, and extracellular pH (pH_e_) from an MRSI method called Biosensor Imaging of Redundant Deviation in Shifts (BIRDS). Spatiotemporal patterns of these parameters during tumorigenesis were unique for each tumor. While U87 tumors grew faster, F_p_, K^trans^, and v_p_ increased with tumor growth in both tumors but these trends were more pronounced for U251 tumors. Perfused regions between tumor periphery and core with U87 tumors exhibited higher F_p_, but K^trans^ of U251 tumors remained lowest at the tumor margin, suggesting primitive vascularization. Tumor growth was uncorrelated with v_e_, ADC, and pH_e_. U87 tumors showed correlated regions of reduced v_e_ and lower ADC (higher cellularity), suggesting ongoing proliferation. U251 tumors revealed that the tumor core had higher v_e_ and elevated ADC (lower cellularity), suggesting necrosis development. The entire tumor was uniformly acidic (pH_e_ 6.1-6.8) early and throughout progression, but U251 tumors were more acidic, suggesting lower aerobic glycolysis in U87 tumors. Characterizing these cancer hallmarks with DCE-MRI, ADC-MRI, and BIRDS-MRSI will be useful for exploring tumorigenesis as well as timely therapies targeted to specific vascular and metabolic aspects of the tumor microenvironment.

## Introduction

Glioblastomas (GBM) are the most frequently occurring and aggressive primary brain tumors in adults (1). Despite intensive surgical and medical intervention, prognoses remain poor with an average survival of less than 2 years (2). Although magnetic resonance imaging (MRI) with transverse (T_2_) or longitudinal (T_1_) relaxation times is used for initial clinical identification upon symptom onset, biopsy remains the gold standard for diagnosis. Specifically, contrast-enhanced T_1_-weighted and T_2_ fluid-attenuated recovery (FLAIR) images are used for clinical detection and size monitoring after treatment (3); however, there remain numerous unmet imaging needs as many findings are nonspecific and do not allow for direct mapping of the tumor microenvironment that may be reflective of treatment response (4). Further, these imaging contrasts largely ignore spatial heterogeneity as tumors are extremely diverse in their anatomic features, do not highlight variation in tumor metabolism and vasculature which are reflective of different genetic mutations in GBM, and are confounded in the setting of different therapies, especially anti-angiogenic therapies, that results in drastic changes in the imaging findings despite little change in the structural tumor architecture (5). Therefore, more specific imaging methods are needed to characterize tumor progression as well as to evaluate therapeutic response. In this study, we aim to demonstrate multi-modal longitudinal characterization of the GBM microenvironment in preclinical GBM models to highlight the spatial heterogeneity that exists in tumor cellularity, vascularity, and metabolism and how it changes throughout tumor progression.

Tumorigenesis in GBMs is highly complex and involves metabolic changes in tumor cells and interactions with the surrounding microenvironment. GBMs, like other solid tumors, display many of the hallmarks of cancer including dysregulated metabolism and decreased vascularization which drives the promotion of angiogenesis (6). Angiogenesis, the development of new blood vessels, occurs in tumors in response to the development of hypoxia and to aid in delivery of nutrients to the metabolically active tumor. However, the newly formed vasculature is tortuous and leaky. The tumor microenvironment is shaped by these abnormalities in the vascular architecture leading to heterogenous and inadequate perfusion.

GBMs are highly heterogeneous due to tumor growth patterns and development of necrosis. Spatial heterogeneity, not only in morphology, but in perfusion and metabolism may contribute to differing patient prognoses and therapeutic efficacy. Detecting and measuring tumor size does not fully capture the tumor phenotype. Hypoxia develops in specific regions of the tumor and contributes to tumor aggressiveness. Therefore imaging methods to quantify the vascular architecture of tumors is important for tumor characterization and has been studied using a magnetic resonance imaging (MRI) technique termed dynamic contrast enhanced (DCE) MRI (7). DCE-MRI models the dynamic uptake and exchange of gadolinium-based contrast agents between the vascular and tissue spaces, specifically those regions in which the agent has extravasated into the extravascular space. This can identify regions of the tumor that have high vascular permeability or conversely, regions that are under-perfused and indicative of regions of hypoxia. The images are acquired with a high temporal resolution and the signal intensity time curves are then converted to a contrast agent concentration term that is then fit to a two-compartment exchange model (8). This allows for several vascular parameters to be quantified, including the plasma flow (perfusion), vascular permeability, extracellular volume fraction, and plasma volume fraction. Changes in tumor vascularity measured with DCE-MRI have been utilized to characterize the efficacy of anti-angiogenic therapeutics (9).

Additionally, GBMs undergo a metabolic upregulation of glycolysis even in the presence of sufficient oxygen, termed aerobic glycolysis (Warburg effect), and once the large amounts of acids made intracellularly are extruded, the extracellular environment is acidified (10, 11). Specifically, extracellular acidosis as defined by a reduction in the extracellular pH (pH_e_) in relation to intracellular pH (pH_i_) is an important indicator of tumor aggressiveness as the reduction in pH_e_ has been directly linked to increasing tumor invasiveness, reduced therapeutic response, increased angiogenesis and immunosuppression (12). In normal brain tissue, the pH_e_ is ~7.2-7.4, but in tumors there is a reduction in pH_e_ to ~6.2-6.8. Metabolic abnormalities are not confined to tumor cells, but also shape the tumor microenvironment leading to distinct locations in the tumor, and perhaps even beyond the tumor boundary, that are more prone to cancer cell survival (13).

Thus, metabolic imaging methods have become a focus in the past decade for tracking cancer. Several imaging methods have been proposed and studied for measuring both pH_i_ as well as pH_e_ (14). While pH electrodes are the gold standard, imaging methods including optical, PET, and MR imaging/spectroscopic methods have been used for quantitative pH mapping. However, these methods vary in their specificity for pH_e_ (vs pH_i_) and their ability to quantify the pH. In this study, a magnetic resonance spectroscopic imaging (MRSI) method is used called Biosensor Imaging of Redundant Deviation in Shifts (BIRDS). BIRDS uses the pH-sensitive chemical shifts of non-exchangeable protons of lanthanide-based contrast agents (15). The chemical shifts are characterized in relation to their temperature and pH sensitivities *in vitro*. The signals are resilient and can be observed even in the presence of strong T_2_ contrast agents and therefore are unaffected by the presence of other contrast agents (16). BIRDS agents have been modified to increase their delivery and thereby their sensitivity (17–19). Further, BIRDS has been used to map the pH_e_ in a variety of tumor models (20, 21).

Quantitative BIRDS-pH_e_ mapping has revealed highly acidic tumors where pH_e_ changes can reflect tumor viability and response to therapy (22, 23). However, BIRDS-pH_e_ mapping requires the use of paramagnetic contrast agents (TmDOTP^5-^) and limitations due to the need for sufficient contrast agent accumulation have previously prevented longitudinal pH_e_ measurements. However, organic anion transporter inhibitors (probenecid) can slow renal clearance of the contrast agent (24). Here we demonstrate that a coinfusion of probenecid and TmDOTP^5-^ can allow for repeated longitudinal pH_e_ mapping in the same tumor over time and when combined with multi-modal imaging can be useful in measuring tumor growth and monitoring the tumor microenvironment in human-derived models of GBM. We were able to measure tumor growth and map pH_e_ in the same U87 and U251 gliomas reproducibly and found that despite significant tumor growth, acidosis plateaus early.

In this study, the cellularity, vascularity, and acidosis of untreated U87 and U251 human-derived GBM models were measured longitudinally throughout tumor progression. U87 and U251 models exhibit many characteristic microenvironmental features of human GBM in different aspects including the development of necrosis, rapid growth, invasion into surrounding tissue, and differences in vascularization that lead to hypoxic regions and enhanced angiogenesis (25, 26). However, a limiting factor in translating preclinical GBM treatment studies is being able to longitudinally and non-invasively map the tumor microenvironment in the same tumor over days and weeks. The vascularization of these tumors is important for delivery of MRI contrast agents including gadolinium agents which are used for the DCE method and TmDOTP^5-^ which is used for BIRDS.

Here we describe a multi-modal MRI study of mapping the vascularity, cellularity, and acidosis in the tumor microenvironment in the same imaging space longitudinally throughout tumor progression, and thus were able to interrogate both spatial and temporal changes of these parameters through tumor progression. We hypothesized that there would be restructuring of the vasculature over time in these models and that metabolism would change throughout progression. While common measures of tumor progression are only tumor size, we hypothesized that these measures would provide additional characterizations of the changing tumor microenvironment that were not solely related to the size of the tumor and changes might develop earlier during tumor formation. Characterization of the vascular and metabolic microenvironment in tumors is essential for choosing therapeutic strategies as well as monitoring therapeutic response.

## Methods

### Cell Culture

U87 and U251 cells were purchased from American Type Culture Collections (ATCC). All cells were maintained in an incubator in a 5% CO_2_ atmosphere at 37°C. Dulbecco’s modified eagle’s medium (DMEM) was supplemented with 10% heat-inactivated fetal bovine serum (FBS) and 1% penicillin-streptomycin. U87 cells were cultured in high glucose (4.5 g/L) DMEM and U251 cells were grown in low glucose (1.5 g/L) DMEM. All cells were used at low passage (<20). Cells (U87 and U251) were harvested at high confluence (>80%) and suspended in serum-free DMEM prior to intracranial injection.

### Animal Models of GBM

All animal protocols were approved by the Institutional Animal Care and Use Committee (IACUC) at Yale University. Female athymic/NUDE rats were acquired from commercial vendors (Envigo) and weighed 150-250g. Animals were maintained in temperature and humidity controlled rooms with food and water provided ad libitum. Animals were housed for a minimum of two weeks prior to experimentation.

For intracranial injection of U87 and U251 cells, animals were anesthetized with isoflurane (2-3%). The animal was positioned in a stereotactic holder and the scalp was sterilized with betadine and 70% ethanol. Local anesthetic (Bupivacaine, 2 mg/kg) was administered prior to a midline incision of the scalp. After exposing the skull, a hole was drilled using a motorized drill at a position 3 mm to the right of the bregma. A 5 µL suspension of 2x10^5^ U87 cells or 5x10^5^ U251 cells was loaded into a 10-µL Hamilton syringe equipped with a 26G needle. The needle was positioned through the burr hole and injected 3 mm below the dura into the right striatum. The cells were injected using a microinfusion pump at a rate of 1 µL/min over 5 minutes. The needle was then left in place for 5 minutes prior to being removed at a rate of 0.5 mm/min to prevent any leakage of cells from the injection site. The burr hole was sealed with bone wax and the incision site was sutured and sterilized. Animals were given carprofen (5 mg/kg, subcutaneously) for analgesia during the procedure which was continued for 48 hours postoperatively. Animals were monitored daily for weight loss or development of any neurological symptoms. Tumors were allowed to grow for 10 days prior to initial MRI experiments to monitor for tumor growth.

### MRI

All imaging data were acquired using an 11.7 T Bruker horizontal-bore spectrometer. 10-15 days after cell injection, animals underwent T_2_-weighted MRI to check for tumor formation. During imaging, body temperature was maintained using a circulating warm water heating pad and Paralube vet ointment was applied to the eyes to prevent dryness. Imaging was repeated every 3-7 days until a tumor was able to be identified on T_2_-weighted imaging, typically 1-2mm, prior to beginning the multi-modal imaging protocol. Due to experimental variations, tumors were initially identified at days 10-28 for U87 tumors and days 13-69 days for U251 tumors. After tumor identification, animals underwent the full multi-modal imaging protocol, which was then repeated every 4-7 days, unless clinical neurological signs of disease progression were evident, in which case the animals were immediately imaged and euthanized. A total of 15 rats with U87 tumors and 13 rats with U251 tumors were used in the study **(** **Supplementary Figure 1**, **Supplementary Table 1** **)**.

For the multi-modal imaging studies, which require the administration of contrast agents, a tail vein infusion line was first established prior to the experiment. During this procedure, the animal was anesthetized with 3% isoflurane and placed on a heating pad to maintain body temperature. A 30G needle was inserted into PE10 (Braintree Scientific, LLC) line and filled with heparinized saline. The needle was inserted into the lateral tail vein of the rat and checked for backflow to ensure correct placement within the vein, prior to being anchored to the tail with tape. Heparinized saline (~50 µL) was flushed through the line approximately every 20 min throughout the remainder of the imaging session to ensure proper functioning of the infusion line and to prevent clotting.

The animal was placed in a prone position within a volume (8 cm) transmit/surface (3.5 cm) receive RF coil. The animal and coil were then placed in the isocenter of the magnet bore. Body temperature was monitored using a rectal fiber-optic temperature probe and the breathing rate was monitored using a respiratory monitor.

The imaging protocol consisted of the following order of sequence acquisition. Positioning and power optimizations were performed using Bruker-defined gradient-echo (GE) and fast spin-echo (FSE) sequences. Shimming was done on an ellipsoid voxel to bring the H_2_O line width down to less than 30 Hz. Quantitative T_2_ mapping was performed by acquiring T_2_-weighted images acquired using a spin-echo sequence with TR 6000ms and 10 TE (10-100ms), FOV 25x25mm^2^, matrix 128x128, and slice thickness 1 mm. Seven coronal slices were chosen that covered the entire tumor volume. Diffusion-weighted imaging was performed using an EPI sequence with 6 b values (0-3000 s/mm^2^). The TR and TE were 3 s and 26.2 ms, respectively, and images were acquired with FOV 25x25mm^2^, matrix 64x64, and slice thickness 1 mm. Three coronal slices were chosen that covered the central portions of the tumor. Apparent diffusion coefficient maps were generated by fitting voxel-level data to a monoexponential function in Matlab. Quantitative T_1_ mapping consisted of T_1_-weighted images acquired using a RARE sequence with TE 10ms, 5 TR (400-8000ms), slice thickness 0.7mm with 0.3mm gap, 7 slices, 25 × 25 mm^2^ FOV, 128 × 128 matrix. Quantitative T_1_ maps were generated by fitting voxel-level data to a monoexponential function in MATLAB. The multi-T_R_ T_1_ sequence was then repeated after 0.25 mmol/kg gadobutrol administration to serve as a post-contrast image which was used, along with T_2_ images, to delineate tumor boundaries. As this was acquired at the end of the DCE acquisition, it represented late (20 min after contrast agent infusion) contrast enhancement. A contrast enhanced T_1_-weighted 3D FLASH was also acquired with TE 5ms, TR 30ms, and 0.33mm isotropic resolution for high-resolution coverage of the entire tumor for coregistration.

### DCE-MRI

DCE-MRI was acquired using a dual-echo spoiled gradient echo acquisition (TR/TE: 39ms/2.5-5ms) every 5 seconds over 22 min. At 2 min into the dynamic scan, a bolus IV injection of 0.25mmol/kg gadobutrol, a clinically available macrocyclic gadolinium agent, was manually injected and then flushed with heparinized saline. Other imaging parameters included a flip angle of 15° and one average for a temporal resolution of 5 s. Three central slices of the tumor were chosen with the exact same in-plane positioning, FOV (25 × 25 mm^2^), and matrix (128 × 128) to be co-registered to the T_1_ data. The slice offset was chosen so that the three slices corresponded to either slices 2-4 or 3-5 of the anatomical imaging.

Measurements from pre-contrast T_1_-weighted images were used to transform the signal time-intensity curves into time-concentration curves (TCCs). The arterial input function (AIF) was measured by collecting arterial blood samples at discrete time points post-injection in a representative animal **(** **Supplementary Figure 2** **)**. A hematocrit of 0.45 was assumed for deriving the contrast agent concentration in the blood. The raw AIF was fit to a bi-exponential curve and applied to the analysis for all datasets. A region of interest (ROI) was placed in the tumor, including the contrast-enhancing rim, which was the area used for all analyses.

To measure vascular parameters [K^trans^ (volume transfer coefficient, min^-1^), F_p_ (plasma flow rate, min^-1^), v_e_ (extracellular volume fraction, unitless), v_p_ (plasma volume fraction, unitless)], a two-compartment exchange model (2CXM) was used to fit the DCE data. The parameters were estimated by fitting each voxel using least squares regression using prior software (27). All parameters were quantified on a voxel by voxel basis and spatially plotted in Matlab overlaid on T_2_-weigheted images.

### BIRDS pH_e_ Measurements

Prior to the start of pH mapping, the animal was removed from the magnet bore and transferred to a 1.5 cm single loop surface coil. The animal was repositioned in a prone position such that the tumor was located where coil sensitivity was highest and then the animal and coil were reinserted into the isocenter of the magnet bore. Position and optimization scans were performed as described previously. Next, quantitative T_2_ mapping was performed for tumor localization. For BIRDS pH_e_ measurements, 100 mg/kg probenecid was administered over 10 min and followed after 20 min by a coinfusion of 100 mg/kg probenecid and 1 mmol/kg TmDOTP^5-^. Probenecid was used to increase the plasma concentration of the circulating contrast agent and reduce its rate of renal clearance. All infusions were performed using a syringe pump at a rate of 15 µL/min for a total infusion time of 100 min. Chemical shift imaging began 45 min after the start of the infusion and was performed to measure the chemical shifts of the pH_e_-dependent proton resonances of TmDOTP^5-^ to calculate pH_e_ within each voxel at an isotropic resolution of 1x1x1mm^3^ as previously described (15, 22, 28). A 205 μs dual-banded Shinnar-Le Reux pulse with a bandwidth of 40kHz was used for excitation of the H2, H3, and H6 resonances of TmDOTP^5-^. The pH_e_ in each voxel was quantified and the average pH_e_ of all voxels within the tumor boundary defined by T_1_ contrast enhancement was measured. At the end of the BIRDS experiment, post-contrast quantitative T_2_ mapping was performed such that reductions in T_2_ could be related to contrast agent concentration as well as T_2_ contrast could be used for identifying tumor margins.

### Histology

At the end of the imaging experiment, animals were perfused and the brain was extracted and fixed in 4% paraformaldehyde prior to slicing into 3 mm coronal slabs. The tissue was paraffin embedded and sectioned into 10 µm slices. Sections were deparaffinized and rehydrated prior to hematoxylin and eosin (H&E) staining using standard protocols. In a subset of tumors, tissue sections also underwent immunohistochemistry (IHC) for proliferating cell nuclear antigen (PCNA) and vascular endothelial growth factor (VEGF) with hematoxylin as a counterstain. In brief, sections were deparaffinized and rehydrated prior to antigen retrieval and peroxidase blocking. Sections were incubated with primary antibodies (PCNA, VEGF) followed by incubation with a biotinylated secondary antibody and 3,3-diaminobenzidine (DAB). All stained sections were mounted on glass slides and microscopy was performed (4×, 10×) for digitization and visualization using an automated high-resolution microscope with stitching capabilities (Keyence).

### Statistical Analysis of Parametric Maps

Each parameter was assessed within the tumor boundary defined by both T_2_ and T_1_ contrast enhancement at each time point. Average tumor values were quantified as the average of all tumor voxels over three imaging slices. Values in all cases are reported as mean ± standard deviation. All statistical analyses were performed in Prism 9 (GraphPad, San Diego, CA). Statistical comparisons between groups were performed using unpaired two-tailed t-tests with normality confirmed using the Shapiro-Wilk test. Otherwise, the Mann-Whitney test was used. Parametric maps were registered in the same space and voxel level distributions were shown to reflect the heterogeneity in parameter measurements.

For the spatiotemporal analysis of each parameter, the imaging slice with the largest tumor cross-sectional diameter was selected. The total number of voxels within the defined tumor boundary was used as a measure of tumor volume, with each voxel representing a volume of 0.04 mm^3^. Each voxel was also assigned a depth value based on distance from the tumor margin, within the 2-dimensional plane of the selected slice. The depth values for voxels of each tumor were individually rescaled so that the largest depth for each tumor would be 1, and this rescaled parameter was defined as the distance to the tumor margin (0 is tumor margin or periphery, 1 is center of tumor). This normalized distance was used to define separate tumor regions such as the tumor margin/periphery (normalized distance: 0-0.25), tumor midsection (normalized distance: 0.26-0.74) and the tumor core (normalized distance 0.75-1). Various relaxation, diffusion, DCE and pH_e_ parameters were plotted against distance from tumor margin and tumor volume. Fitting was done using quadratic surfaces (2^nd^ order polynomials). Given internal tumor heterogeneity, the Bisquare fitting algorithm was used to make the fitting robust to outliers. For easier comparison, coefficients for the polynomial surface fit were normalized to the average parameter value and the largest tumor volume.

## Results

### Tumor Growth

**Figure 1:**
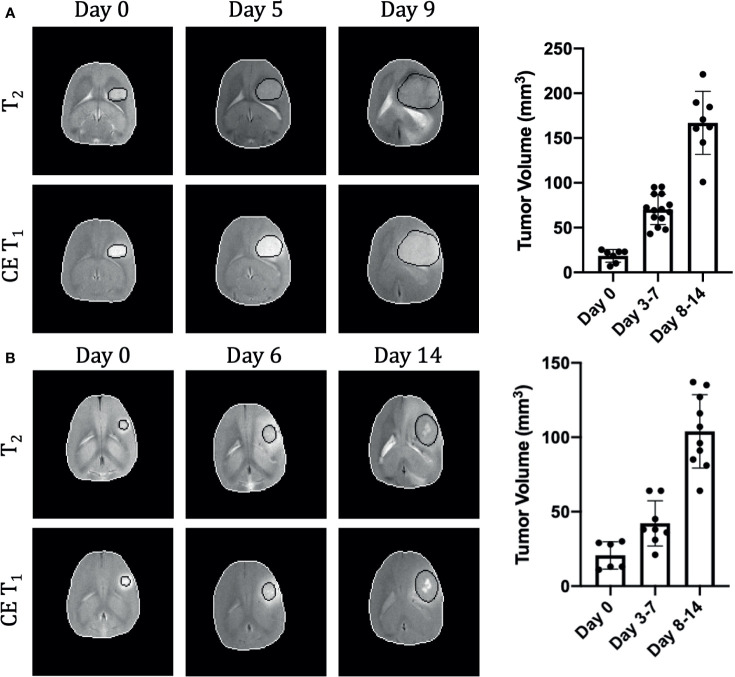
Longitudinal growth characteristics of U87 **(A)** and U251 **(B)** tumors. T_2_ and contrast enhanced T_1_-weighted imaging contrasts are shown at three time points in the same tumor. The tumor is outlined in black and the brain in white. Tumor volume was measured across all imaging slices and quantified by multiplying the number of tumor voxels by the imaging spatial resolution and slice thickness. U87 data includes 29 timepoints from 15 animals and U251 data includes 23 timepoints from 13 animals.

**Figure 2:**
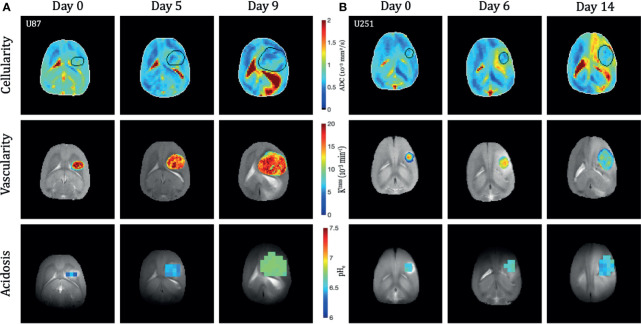
Longitudinal multi-modal imaging of U87 **(A)** and U251 **(B)** tumors. The apparent diffusion coefficient (ADC) maps are shown as a reflection of cellularity, the K^trans^ map from DCE is shown to reflect vascularity, and the pH_e_ map from BIRDS is shown to reflect acidosis. All imaging was performed at three time points in the same tumor. The tumor was masked using T_2_-weighted and contrast-enhanced T_1_-weighted imaging. Vascular parameters from DCE and pH_e_ from BIRDS were calculated in the masked tumors.

Both U251 and U87 tumors were monitored longitudinally over up to three time points. A total of 29 time points were acquired across 15 rats with U87 tumors and 23 time points acquired across 13 rats with U251 tumors **(** **Supplementary Table 1** **)**. Only data sets in which all imaging contrasts were successfully obtained were included in the analysis and included 24 timepoints for U87 tumors and 17 timepoints for U251 tumors **(** **Supplementary Figure 1** **)**. Tumor volume was measured using both T_2_-weighted and contrast-enhanced T_1_-weighted imaging to manually identify tumor margins over all imaging slices **(** **Figure 1** **)**. The segmentation of tumors appeared differently in U251 tumors compared to U87 tumors due to the presence of peritumoral edema. In U251 tumors, the surrounding edema appeared brighter on T_2_-weighted images in comparison to the tumor. The darker appearance of the tumor was selected for tumor volume measurements to ensure consistency. U87 tumors were uniformly contrast enhancing which was used for tumor margin demarcation and volume measurements. After tumor detection, size increased exponentially in both U251 and U87 tumors **(** **Figure 1** **)**. Tumor growth was normalized to when tumors were first identified by imaging (Day 0), usually when tumor volumes were less than 30 mm^3^. The tumor size at time of initial scan was 18.4 ± 7.1 mm^3^ for U87 tumors and 20.7 ± 9.2 mm^3^ for U251 tumors. By the final time point, tumor volume reached 166.9 ± 35.2 mm^3^ for U87 tumors and 103.9 ± 24.6 mm^3^ for U251 tumors. While at the initial time point the two tumors were approximately of the same size, at the final time point both tumors grew significantly larger but U87 tumors were ~60% greater than U251 tumors (p<0.0004). At the same time points, cellularity (from ADC), vascularity (from DCE), and metabolism (from BIRDS) were also successfully monitored longitudinally in the same subjects **(** **Figure 2** **)**. All average values reported for ADC, DCE parameters and pH_e_ are an average value across all voxels identified as tumor. Further demonstration of the distribution and heterogeneity of parameter values are also reported in a histogram analysis **(** **Supplementary Figure 3** **)**.

### MRI Relaxation [T_1_, T_2_, and Contrast-Enhanced (CE) T_1_]

**Figure 3:**
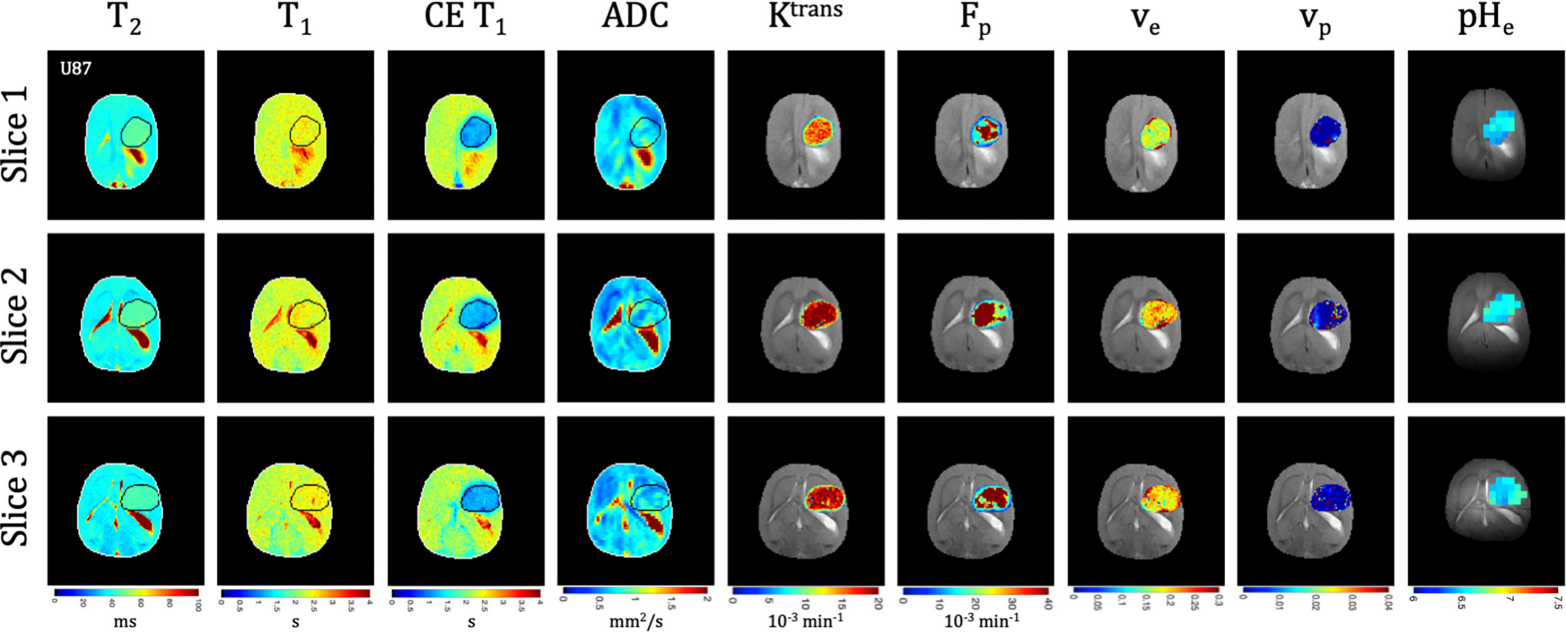
Multi-slice, multi-parametric imaging of U87 tumors. All data is from a single imaging session for a single animal. Cellularity (ADC), vascularity (F_p_, K^trans^, v_e_, v_p_ from DCE) and metabolism (pH_e_ from BIRDS) as well as quantitative T_2_ and pre/post-contrast T_1_ mapping are included. Shown are three imaging slices that were used to acquire all imaging contrasts allowing for extensive 3D characterization of the tumor microenvironment. Multi-slice measurements were performed for all included time points.

**Figure 4:**
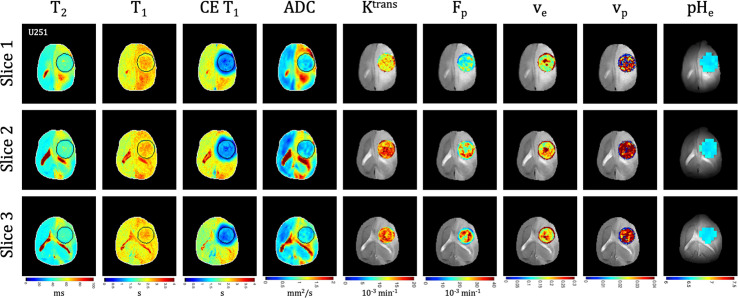
Multi-slice, multi-parametric imaging of U251 tumors. All data is from a single imaging session for a single animal. Cellularity (ADC), vascularity (F_p_, K^trans^, v_e_, v_p_ from DCE) and metabolism (pH_e_ from BIRDS) as well as quantitative T_2_ and pre/post-contrast T_1_ mapping are included. Shown are three imaging slices that were used to acquire all imaging contrasts allowing for extensive 3D characterization of the tumor microenvironment. Multi-slice measurements were performed for all included time points.

Quantitative T_1_ and T_2_ relaxation maps were acquired at multiple timepoints in addition to the advanced vascular and metabolic imaging methods. In both U87 **(** **Figure 3** **)** and U251 **(** **Figure 4** **)** tumors, T_1_ and T_2_ values were higher in the tumor compared to surrounding tissue. For T_1_ the values between tumors were not significantly different at the final time point (p=0.22), but T_1_ increased with tumor progression from the initial to final timepoint for both tumors (U87: 2.24 ± 0.13 s *vs*. 2.53 ± 0.07 s, p<0.0001; U251: 2.19 ± 0.09 s *vs*. 2.47 ± 0.04 s, p=0.0009). For T_2_ the values between tumors were moderately different at the final time point (p=0.026), but there was a trend in increasing T_2_ with tumor progression for both tumors (U87: 41.3 ± 2.9 ms *vs*. 43.3 ± 2.1 ms, p=0.16; U251: 38.5 ± 2.5 ms *vs*. 41.0 ± 1.5 ms, p=0.06).

Tumors displayed signal enhancement (CE T_1_) and significantly reduced T_1_ values within the tumor core after gadobutrol infusion on T_1_-weighted images in U87 **(** **Figure 3** **)** and U251 **(** **Figure 4** **)** tumors. For CE T_1_, the values between tumors were not significantly different at the final time point (p=0.26), but CE T_1_ increased with tumor progression for U87 (1.12 ± 0.10 s *vs*. 1.29 ± 0.16 s, p<0.03) but not U251 (1.27 ± 0.20 s *vs*. 1.16 ± 0.25 s, p=0.43). U87 tumors displayed homogenous signal enhancement that was confined by the tumor margins **(** **Figure 3** **)**; however, U251 tumors exhibited much greater heterogeneity in enhancement patterns with contrast accumulating in central tumor regions as well as leaking into surrounding brain tissue resulting in contrast enhancing regions changing based on the duration after contrast agent administration **(** **Figure 4** **)**. These patterns were reflected in post-contrast quantitative T_1_ mapping such that with tumor progression, U87 tumors had higher post-contrast T_1_-values than U251 tumors which could reflect decreased uptake of contrast in these tumors as well as the same amount of contrast agent distributing in a larger tumor volume resulting in decreased concentration and lesser T_1_ shortening.

### ADC and DCE

Quantitative ADC maps were generated across 3 imaging slices at each time point from diffusion-weighted imaging at multiple b values. Since ADC has been shown to be correlated with tumor cellularity (29), the ADC was used as an indication of the proliferative status of U87 **(** **Figure 3** **)** and U251 **(** **Figure 4** **)** tumors. ADC parameters were distributed over a narrow range and trended towards lower values with progression across both tumor types. However, the ADC values between tumors were not significantly different at the final time point (p=0.26), nor did they change for either tumor between time points (p=0.72 for U87; p=0.75 for U251) suggesting similar cellularity between U87 and U251 tumors.

There was heterogeneous dynamic uptake of gadobutrol within the tumor core as measured with DCE and reflected in the vascular parameters. Further, with tumor progression there was increased heterogeneity in the vascular parameters. F_p_ and K^trans^ were lowest at the tumor periphery in both U87 **(** **Figure 3** **)** and U251 **(** **Figure 4** **)** tumors. For all DCE parameters the values between tumors were not significantly different at the final time point, nor did they change for either tumor between time points **(** **Supplementary Table 2** **)**. However, the parameter values were highly heterogenous within each tumor which leads to the large standard deviations. At the final time point, there also was no difference between the tumor types for K^trans^ (p=0.33), v_e_ (p=0.30), and v_p_ (p=0.73); however, the perfusion (F_p_) tended to be higher in U87 tumors although this did not reach significance (p=0.08).

### BIRDS

Signal enhancement after gadobutrol administration corresponded to regions of signal darkening after TmDOTP^5-^ in T_2_-weighted images providing multiple contrasts for measuring tumor size. The SNR achievable with BIRDS showed TmDOTP^5-^ uptake in the tumor and peritumoral regions with little agent in the normal brain **(** **Supplementary Figure 4** **)**. Further, coinfusion of probenecid and TmDOTP^5-^ allowed for successful repetitive pH_e_ mapping in the same tumor for the first time. At all time points, the intratumoral pH_e_ was lower (pH 6.1-6.8) than the pH_e_ of the brain parenchyma (~7.1) indicating that the intratumoral microenvironment becomes acidic early during tumor growth, once the tumor becomes detectable by imaging methods. However, longitudinal pH_e_ mapping using BIRDS revealed as tumors become larger there is no clear corresponding change in pH_e_ such that acidosis developed early during tumor formation and remained acidic throughout tumor progression. Across all timepoints, the average pH was higher in U87 compared to U251 (6.54 ± 0.08 *vs*. 6.47 ± 0.14; p=0.046). In U87 tumors, larger tumors had the same pH_e_ as smaller tumors (6.56 ± 0.05 *vs*. 6.56 ± 0.09, p=0.94); however, in U251, larger tumors tended to be more acidic (6.44 ± 0.15 *vs*. 6.58 ± 0.06, p=0.12). The pH_e_ remained relatively homogenous throughout the tumor with minimal spatial variation. The pH_e_ at the core of both tumors reached similar values in smaller tumors; however, in U87 tumors, the pH_e_ initially decreased with progression, but was then higher in the largest tumors.

### Spatiotemporal Patterns of Tumorigenesis

**Figure 5:**
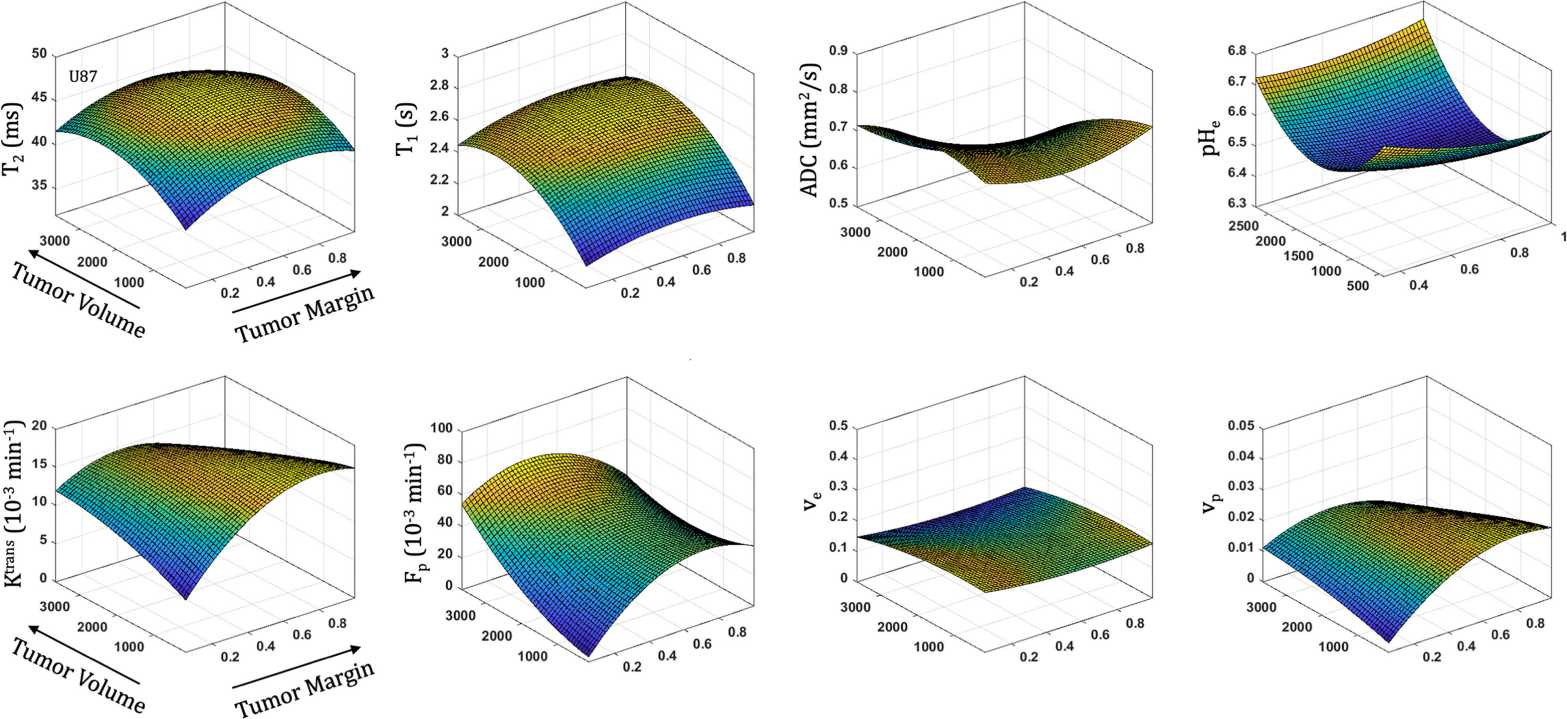
Spatial and temporal variation in parameter measurements throughout U87 tumor progression. With progression, tumor volume increases. The spatial distribution is reflected as distance from tumor margin (0 is tumor margin, 1 is center of tumor). Cellularity (ADC), vascularity (F_p_, K^trans^, v_e_, v_p_ from DCE) and metabolism (pH_e_ from BIRDS) as well as quantitative T_2_ and pre/post-contrast T_1_ parameter values are included. All imaging voxels across all timepoints and all tumors were fit to a 2^nd^ order polynomial to illustrate the changing spatial parameter distributions with tumor progression.

**Figure 6:**
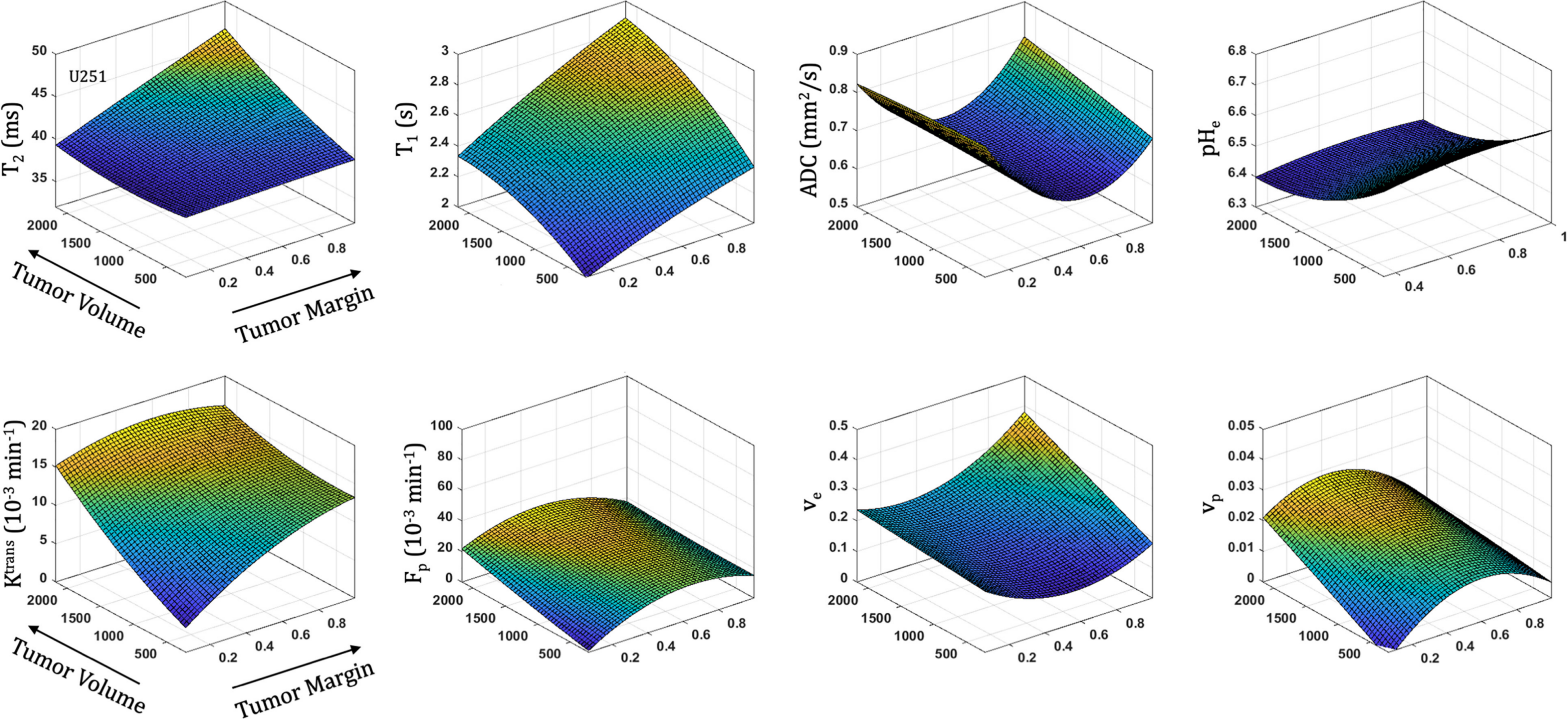
Spatial and temporal variation in parameter measurements throughout U251 tumor progression. With progression, tumor volume increases. The spatial distribution is reflected as distance from tumor margin (0 is tumor margin, 1 is center of tumor). Cellularity (ADC), vascularity (F_p_, K^trans^, v_e_, v_p_ from DCE) and metabolism (pH_e_ from BIRDS) as well as quantitative T_2_ and pre/post-contrast T_1_ parameter values are included. All imaging voxels across all timepoints and all tumors were fit to a 2^nd^ order polynomial to illustrate the changing spatial parameter distributions with tumor progression.

All parameters were quantified with increasing distance from the tumor margin (spatial) and tumor volume (temporal) in both U87 **(** **Figure 5** **)** and U251 **(** **Figure 6** **)** tumors. Coefficients for the polynomial surface fit of each 3D spatiotemporal plots for both U87 and U251 tumors were generated from quadratic fits which were superior to linear fits to the 3D surface.

**Figure 7:**
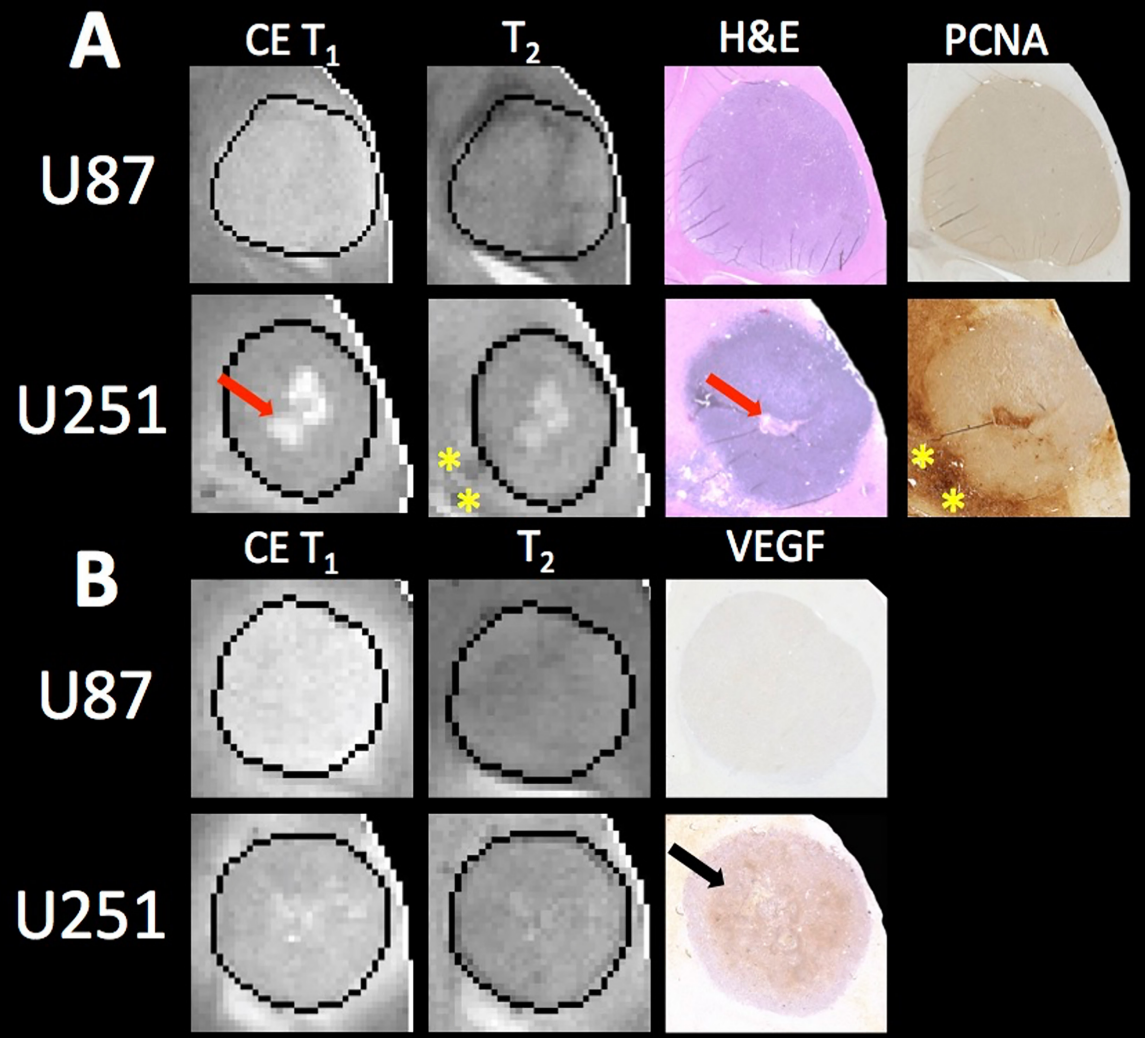
MRI and pathology comparisons for U87 and U251 tumors. **(A)** Contrast enhanced (CE) T_1_-weighted and T_2_ weighted MRI along with H&E staining and PCNA IHC are shown for example U87 and U251 tumors. Necrotic regions (red arrows) form in the central regions of U251 tumors as evident on H&E, which corresponds to regions of higher signal intensity on T_1_-weighted images where contrast agent accumulates. Necrosis was not observable in U87 tumors. Regions of increased proliferation (yellow stars) are highest on the tumor margins and extend into surrounding tissue in U251 tumors. **(B)** MRI with corresponding VEGF IHC in U87 and U251 tumors. Increased VEGF was observed in mid-regions of U251 tumors (black arrow).

With increased tumor progression (increasing volume), there was an increase in intrinsic T_1_ in both U87 and U251 tumors. However, there was a homogenous distribution of the T_1_ and T_2_ relaxation measurements across U87 tumors independent of tumor size. U251 tumors exhibited increases in T_1_ and T_2_ in central regions of larger tumors that were not evident at initial tumor detection, possibly indicative of necrotic cores that were not evident in U87 tumors as supported by H&E histological findings **(** **Figure 7A** **)**, where necrotic regions are indicated by red arrows in U251 tumors and no necrotic regions were observed in U87. Additionally, the spatial distribution of ADC values was not uniform, with higher ADC values observed at the tumor margin and in the center of the tumor with these differences being more pronounced in U251 tumors. The highest values of ADC were observed in central tumor regions of large U251 tumors, the same regions that exhibited higher T_1_ and T_2_ relaxation rates. The spatiotemporal patterns of changes in ADC were similar in both U87 and U251 tumors as ADC was higher at the periphery regardless of tumor size, as supported by PCNA IHC data **(** **Figure 7A** **)** with increased PCNA staining at the tumor margins.

In U87 tumors, there was much greater homogeneity in the vascular parameter values. The extracellular volume fraction and plasma volume fraction tended to decrease with tumor progression. However, the highest value in K^trans^, F_p_, and v_p_ were seen in midregions of the tumor (lower in central regions of the tumor and the periphery). Tumor progression led to extracellular volume (v_e_) reduction as well as an increase in plasma flow (F_p_); however, vascular permeability (K^trans^) remained constant. Although the average vascular permeability did not change, there was an increase in heterogeneity such that regions with higher vascular permeability also had higher plasma volume fraction and plasma flow.

In U251 tumors, there were notable differences in the extracellular volume (v_e_) fraction with tumor progression. With tumor progression, the extracellular volume fraction (v_e_) increased significantly in central regions of the tumor. This corresponded with a reduced plasma fraction (v_p_) and perfusion (F_p_) in central regions of the tumor. In U251 tumors, the vascular permeability (K^trans^) increased with tumor progression and the spatial heterogeneity actually decreased, reflective of higher K^trans^ values throughout the tumor and as supported by increased VEGF IHC staining throughout the tumor **(** **Figure 7B** **)**.

At the time of initial tumor detection, when tumors were small, permeability and the extracellular volume fraction (v_e_) were comparable between U87 and U251 tumors and showed increasing values with tumor progression that was most pronounced at the tumor margin. In both U87 and U251 tumors, F_p_ exhibited similar increasing trends during tumor progression, but at all stages of tumor growth, perfusion tended to be higher in U87 tumors. Additionally, the plasma volume fraction increased at the periphery of both U87 and U251 tumors with tumor progression.

## Discussion

U87 and U251 tumors were characterized in terms of their cellularity, vascularity, and metabolism throughout tumor progression with multi-parametric MRI/MRSI methodology. Few longitudinal studies on preclinical glioma models have been performed and normally focus only on one additional imaging contrast (30–32). The pH_e_ has previously been monitored longitudinally in C6 glioma models and found only small changes in the pH_e_ with tumor progression (33). In this prior study, pH_e_ was measured using hyperpolarized bicarbonate and the intracellular pH was measured using a ratiometric chemical exchange saturation transfer (CEST) approach termed AACID. Here we show that pH_e_ in both U87 and U251 tumors also remained relatively stable throughout tumor growth. The acidic microenvironment, with pH_e_ values ranging from 6.1 to 6.8, developed by the initial timepoint early during tumor progression and remained homogenous. However, U251 tumors were found to be slightly more acidic, suggesting that U87 tumors are metabolically less glycolytic.

During tumor progression, spatiotemporal patterns of parametric values were distinct for both U87 and U251 tumors, indicating differences in tumor architecture, vascularity, and metabolism. Unique tumor growth patterns were evident between U87 and U251 tumors, with U87 tumors growing to twice the tumor volumes of U251 tumors at the final time point. While F_p_, K^trans^, and v_p_ increased with tumor growth, these changes were more pronounced for U251 tumors. Although K^trans^ remained lowest at the tumor margin of both tumors, well-perfused regions developed in central tumor regions between the periphery and core with U87 tumors exhibiting higher F_p_. Although there was no change in average values for v_e_ and ADC with progression, regions with reduced v_e_ corresponded well with lower ADC (higher cellularity) in U87 tumors and for U251 tumors, regions with higher v_e_ and higher ADC were observed in the core suggesting development of necrosis.

### Tumor Growth Patterns

Despite U87 and U251 tumors being extensively used in the literature, different growth patterns are evident in many reports (34–36). U251 is a more proliferative and infiltrative tumor in comparison to U87; however, both tumor types are angiogenic. Additionally, in U251 tumors, areas of hypoxia and ultimately necrosis develop, whereas U87 tumors do not develop necrosis. Differences in time to tumor formation results from the difficulty of injection large numbers of cells in small volumes. A larger number of cells (5x10^5^ cells in 5 µL) are injected for the U251 model compared to U87 (2x10^5^ cells in 5 µL) and the variation in time to tumor formation is much greater **(** **Supplementary Table 1** **)**. Additionally, U251 tumors do not appear as a uniform mass early during tumor growth, making detection more difficult and therefore the first scan more variable. U251 tumors are also smaller than U87 tumors at the time of final scan due to development of neurological symptoms at smaller tumor volumes likely due to the presence of peritumoral edema.

The size of the tumor based on contrast enhancement depends on the time from contrast agent administration until measurement. Previous work comparing the tumor size from contrast-enhanced imaging revealed the difference in size based on the time from contrast administration with differences observed up to 30% greater at late time points (37). Although late enhancement T_1_-weighted images were acquired, the volume was calculated based on demarcation of the tumor margin on T_2_-weighted images for consistent measurements between the tumor types and to exclude peritumoral edema from the tumor volume measurements. In large tumors, a greater number of slices was required for full 3D coverage of the tumor. For this reason, the 7 slices acquired for the T_2_-weighted images were used for tumor volume measurements. The DCE and pH_e_ data were acquired in three 1 mm slices and although in small tumors would cover the entirety of the tumor, in larger tumors represents the superior and central portions of the tumor. Three slices were chosen for these measurements due to the tradeoff between temporal resolution and amount of data collected in DCE measurements and the pH_e_ measurements using BIRDS becoming SNR limited with increasing distance from the receiver coil (deeper regions of the brain).

### Vascular Measurements

The measurements from DCE-MRI reflect the extent of contrast agent extravasation and washout from the tumor and relate these changes to local perfusion and vascular permeability. Angiogenesis results in the formation of abnormal vasculature with increased vessel wall permeability that is reflected in DCE vascular measurements (38). Both U87 and U251 are well vascularized tumors. Well-perfused regions (F_p_) developed between the periphery and tumor core with U87 tumors exhibiting higher perfusion rates, but permeability (K^trans^) remained lowest at the tumor margin for both tumors. A high extracellular volume fraction (v_e_) in the central regions of U251 tumors reflected the development of necrosis. Additionally, this is correlated with increases in T_2_ and ADC reflective of increased free water that would be present in necrosis. Interestingly tumor progression showed correlated changes between extracellular space (v_e_) and tissue cellularity (ADC). For U87 tumors, regions with reduced v_e_ corresponded well with lower ADC (higher cellularity), whereas for U251 regions with higher v_e_ and higher ADC were seen in the core suggesting development of necrosis.

Histology was used to corroborate the presence of necrosis development in U251 tumors. Central regions of U251 tumors had reduced cellularity as reflected by decreased hematoxylin staining on H&E; however, U87 tumors showed no evidence of necrosis. Necrotic areas on H&E corresponded to regions of higher ADC and increased v_e_ also suggestive of reduced cellularity as expected with necrosis. While both U87 and U251 are positive for PCNA, as validated with IHC, U251 has much greater proliferation at the tumor margin. Further, PCNA is expressed at high levels in peritumoral tissue in U251 tumors indicative of proliferative cells outside of the demarcated tumor boundary in the peritumoral area.

VEGF IHC revealed increased angiogenesis in the mid-regions of U251 tumors that was not seen in U87 tumors; however, the overall staining for VEGF in U87 tumors was faint. VEGF expression is regulated by hypoxia which promotes angiogenesis and an increase in vascular permeability. U87 has higher perfusion throughout the tumor (increased F_p_) and therefore hypoxia does not develop, which is supported by the lack of VEGF staining. However, U251 tumors have lower F_p_ and higher vascular permeability (K^trans^) in mid-regions of the tumor (between the margin and center of the tumor) compared to U87 tumors, which is supported by increased VEGF staining.

### Metabolism

The acidic microenvironment (pH_e_ 6.1-6.8) developed early during tumor progression and remained homogenous with U251 tumors being more acidic, suggesting U87 is metabolically less active. Although the pH_e_ remains stable or slightly increases at late time points in U87 tumors, the volume that is acidified is exponentially larger, indicating significant changes in metabolic output despite the small variations in pH_e_. Given that the pH_e_ remains acidic at the tumor margin, further characterization of the pH_e_ changes in the peritumoral regions may shed insights into parenchymal transformation as well as the role of surrounding stroma on tumor growth. U87 has a well demarcated tumor margin that remains well defined throughout tumor progression indicating minimal invasion into the surrounding tissue. In contrast, U251 tumors have a poorly defined tumor margin where contrast leaks into the surrounding tissue, and ADC and T_2_ values are higher in the peritumoral region, making measuring spatial variations in pH_e_ at the tumor margin important for characterizing tumor invasion into surrounding tissue. However, pH_e_ distributions remain homogenous throughout tumor progression and a stable marker of tumor metabolic output.

### Spatial and Temporal Changes With Tumor Progression

Quadratic surface fitting was used to relate both spatial and temporal variations in parameter values throughout tumor progression and used tumor volume as a longitudinal proxy of progression, rather than time to progression in individual animals. This approach was utilized because determining the starting point for each tumor was infeasible due to variability in initial tumor formation. Additionally, due to tumor heterogeneity, tumor progression did not occur at the same or predictable pace for each animal. Combining data from different animals allowed for the elucidation of more universal and robust trends for the different tumor lines rather than a focus on animal to animal tumor variations.

A surface fitting analysis is a powerful tool as it is able to reveal simultaneous patterns and trends in both the temporal dimension (with tumor progression and increase in tumor volume) as well as the spatial dimension (distance to tumor boundary). However, the extraction of fitted surfaces from any given data comes at the potential tradeoff of overfitting, thus requiring optimal design choices. One of these was the degree of polynomial fitting, with second-degree (quadratic) surfaces chosen to capture consistent relationships across each tumor type and parameter values. The lack of any discernable higher order patterns made any higher order polynomials (cubic or higher) susceptible to overfitting and inappropriate for this analysis.

Our data, understandably, is not a random sampling of voxels but rather contains many imaging voxels from tumors that were acquired at multiple times during tumor growth. Since data from multiple timepoints across tumors were used, voxels from each tumor would naturally show some correlation as there are more datapoints (voxels) across a range of distances from tumor boundary than across a range of tumor volumes. The result of this is that trends along the “distance to tumor boundary” will be more robust than trends along the tumor progression/tumor volume axes, and it should also be noted that quadratic fitting could be skewed by an outsized impact from tumors that are both of unusually large size and that violate the normal tumor line trends. While this was a potential limitation, visual inspection of the data did not indicate this to be the case and showed good interpolation between tumor volumes, and argues for the merits of the analysis which has been possible here.

Given tumor heterogeneity, and the presence of small amounts of “outlier” tissues such as blood vessels, we used a robust automatic outlier weighting regression algorithm (Bisquare) for our fitting. However, to check robustness of the choice of outlier removal, we also analyzed the data with the least absolute residuals (LAR) regression algorithm resulting in trends that did not change, giving confidence that our outlier algorithm did not introduce any processing artifacts into the results.

### Limitations

Several previous limitations of the BIRDS method for measuring pH_e_ were overcome in this study. For the first time, pH_e_ is reported at multiple depths within the tumor from the 3D dataset allowing for spatial pH_e_ characterization throughout a larger region of the tumor. Additionally, while we have previously shown that the use of probenecid did increase tissue concentrations of TmDOTP^5-^ (24), this study presents the first application of longitudinal pH_e_ mapping using BIRDS. While probenecid has some buffering capacity, the total amount injected does not affect blood pH values. Although the ADC/DCE and BIRDS datasets were acquired in different coils, animal positioning was standardized to ensure that the same regions were imaged in both datasets and were acquired in the same imaging session. The slices used from the BIRDS dataset were acquired with the same thickness as for the ADC and DCE measurements such that they represent similar tumor regions. Although minor rotations in the animal positioning prevent the slices acquired from the two methods to be considered exact, they represent the same tumor depths and spatial orientation. The tumor was masked independently in the DCE and BIRDS dataset to account for these small discrepancies in positioning. Future use of a radio frequency coil design that optimally captures the DCE and BIRDS data would resolve these concerns.

Additional analytic methods could be applied to evaluate tumor regions either on a voxel-level basis or using a cluster analysis. Voxel wise correlations are possible when considering grouped tumor sizes **(** **Supplementary Figure 5** **)**; however, it does not allow for tracking of specific tumor regions longitudinally as it is difficult to register voxels in the growing tumor across time. Additionally, further analysis with image coregistration between imaging datasets and histological slices is more precisely needed for greater histopathological correlation. While probenecid is used clinically, TmDOTP^5-^ is not approved for clinical use. However, transition metal probes with similar pH sensitivity are being developed to increase translatability. Additionally, BIRDS can be used in preclinical models to validate other pH sensing methodologies. Further, BIRDS has recently been implemented on clinical 3T MRI scanners using a rabbit model of liver cancer (20, 21) showing that the method is translatable to clinical MR scanners.

As with all preclinical work using stable cell lines, inherent limitations reflect the fact that malignant cells are injected intracranially. Therefore, the earliest stages of tumorigenesis are not reflected and metabolic reprogramming is likely already present in such cell lines. Future studies utilizing *in vivo* malignant transformation or animals with genetic predisposition to tumor formation may be considered in future preclinical work.

## Conclusion

Vascular, cellular, and metabolic imaging provides rich information on the microenvironment during tumor progression in GBM. For example, although U87 tumors grew two times faster, U251 tumors were more aggressive as indicated by their prolonged and reduced perfusion and pH_e_ as well as development of necrotic cores. In addition to changing morphologic architecture and cellularity, the tumor vascular microenvironment showed a reduction in extracellular volume, increased plasma flow, and greater heterogeneity throughout tumor progression. Using a combination of probenecid and TmDOTP^5-^ allows for successful longitudinal BIRDS pH_e_ quantification. Interestingly, despite the morphologic and vascular remodeling, metabolic output remains stable and equivalently glycolytic as evident by constant pH_e_, with the acidic microenvironment developing early in tumor growth and remaining acidic during tumor progression, possibly providing a biomarker of tumor metabolism that is independent of tumor size and may in future studies be used to monitor treatment response. The dynamic processes regulating transformation of the tumor microenvironment suggests tumor characteristics that must be considered for targeted therapies and where the homogeneity of pH_e_ during tumor progression may prove useful for evaluating therapeutic response in the context of heterogeneous and changing relaxation, diffusion, and vascular parameters.

## Data Availability Statement

The raw data supporting the conclusions of this article will be made available by the authors, without undue reservation.

## Ethics Statement

The animal study was reviewed and approved by the Institutional Animal Care and Use Committee of Yale University.

## Author Contributions

JW, DC, and FH designed the study. JW, MP, LA, SM, SKM, and MH conducted experiments. JW, MP, and AA analyzed data. All authors contributed to the article and approved the submitted version.

## Funding

This work was supported by NIH R01 EB-023366 and T32 GM-007205.

## Conflict of Interest

The authors declare that the research was conducted in the absence of any commercial or financial relationships that could be construed as a potential conflict of interest.

## Publisher’s Note

All claims expressed in this article are solely those of the authors and do not necessarily represent those of their affiliated organizations, or those of the publisher, the editors and the reviewers. Any product that may be evaluated in this article, or claim that may be made by its manufacturer, is not guaranteed or endorsed by the publisher.

## Supplementary Material

Supplementary Table 1 Imaging timepoints for individual U87 and U251 tumors. A total of 15 animals bearing U87 tumors and 13 animals bearing U251 tumors were imaged. Animals were imaged up to three times during tumor progression, based on tumor growth kinetics and development of neurological symptoms. The tumor volumes corresponding to each imaging timepoint are also included as reflected in **Figure 1**.

Supplementary Table 2 Quantitative values and statistical comparisons for K^trans^, F_p_, v_e_, and v_p_ vascular parameter values from DCE-MRI for U87 and U251 tumors between the initial and final time point. Parameter values were averaged across all 3 slices from all tumors and are reported as average ± standard deviation. Two tailed t tests are used to compare changes in the parameter values with tumor progression.

Supplementary Figure 1 Experimental design. Animals were inoculated with either U87 or U251 cells and tumors were allowed to grow. Starting 10 days after inoculation, the animals were scanned to identify the presence of tumor. These scans were repeated every 4-7 days until tumors were identified. After tumor identification, approximately 1-3 days later the first longitudinal scan was performed (Day 0, timepoint 1). These scans were then repeated on day 3-7 and day 8-14. Only data sets in which all imaging modalities were completed (ADC, DCE, BIRDS) were included in the final analysis.

Supplementary Figure 2 AIF used for DCE analysis. The AIF was based on normalized blood contrast agent concentrations obtained from manual arterial blood sampling during gadobutrol infusion in a representative animal (red dots). This was compared with signal intensity measurements taken from the sagittal sinus of the same animal (blue dots). The AIF was fit to a biexponential function **(B)** which was then utilized for all DCE analysis.

Supplementary Figure 3 Histogram analysis for all parameters measured in U87 and U251 tumors. All tumor voxels are grouped based on the three timepoints and shown as a distribution of parameter values with red being the first timepoint (small tumors), blue the second timepoint, and green the third timepoint (large tumors).

Supplementary Figure 4 MRSI with BIRDS. Voxel-level spectra of TmDOTP^5-^ demonstrating three sharp peaks corresponding to three distinct proton resonances in U87 **(A)** and U251 **(B)** tumors. The data show that the agent is primarily located within the tumor with decreasing concentrations in the peritumoral regions, especially in U251 tumors. There is minimal agent accumulation in the normal-appearing brain.

Supplementary Figure 5 Voxel-level analysis for U87 tumors. Measurements for individual voxels could be compared across multiple different parameters. The fraction of voxels with each parameter value were included from all tumors at each timepoint. These relationships demonstrate similar findings to the histogram analysis with increasing distribution of parameter values at later imaging time points (larger tumor volumes). The pH_e_ measurements are not included here because although they were acquired from the same animal, they were acquired with slightly different positioning and at larger voxel sizes, making direct voxel-level correlations with the other parameters challenging.
