# Supplementary figures and images for: Imaging Hallmarks of the Tumor Microenvironment in Glioblastoma Progression
Source: Front Oncol. 2021 Aug 26;11:692650. doi: 10.3389/fonc.2021.692650 (PMC8426346; doi:10.3389/fonc.2021.692650)

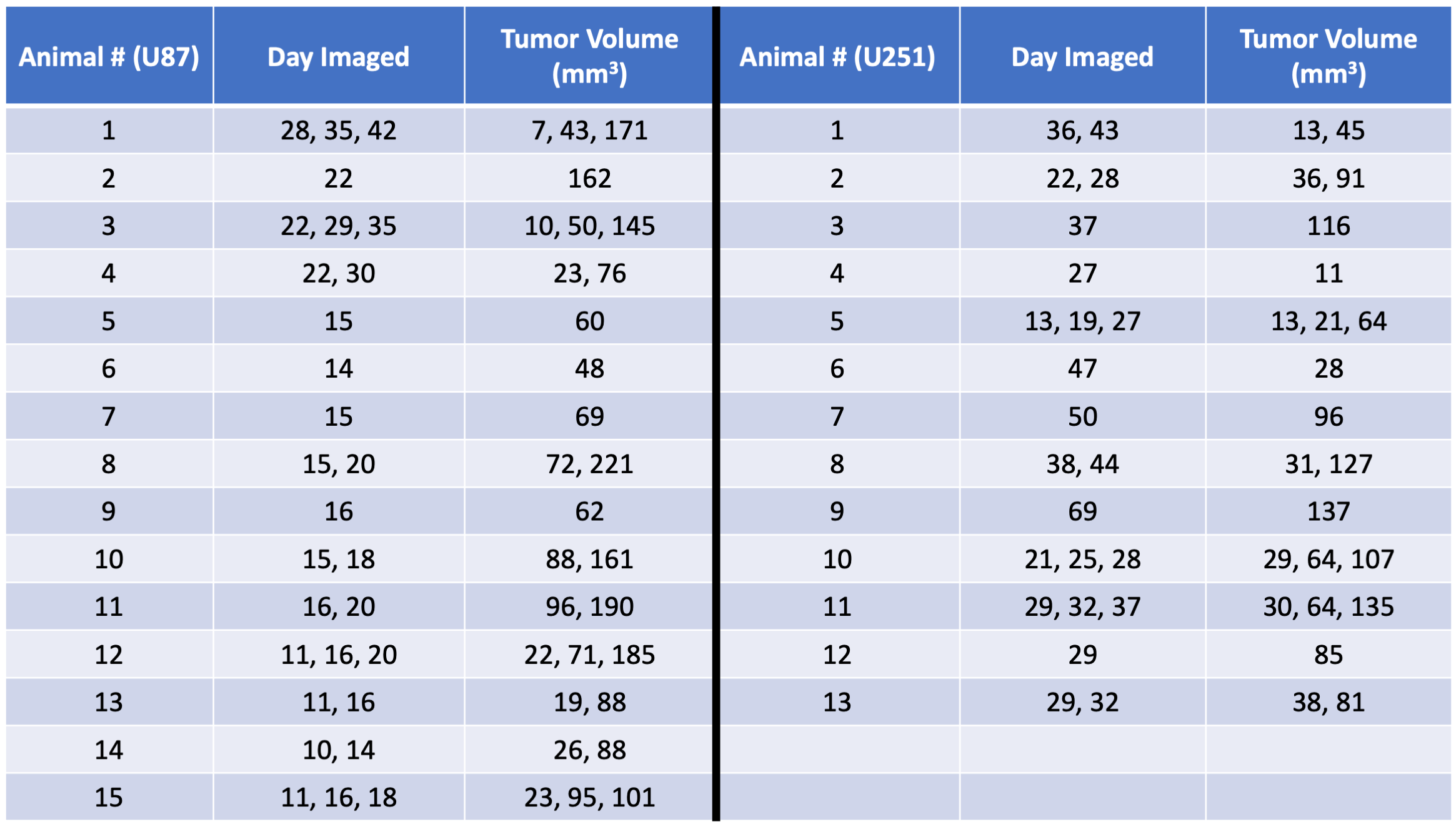

Supplement: Supplementary Table 1 — Imaging timepoints for individual U87 and U251 tumors. A total of 15 animals bearing U87 tumors and 13 animals bearing U251 tumors were imaged. Animals were imaged up to three times during tumor progression, based on tumor growth kinetics and development of neurological symptoms. The tumor volumes corresponding to each imaging timepoint are also included as reflected in Figure 1. [file Image_1.tiff]

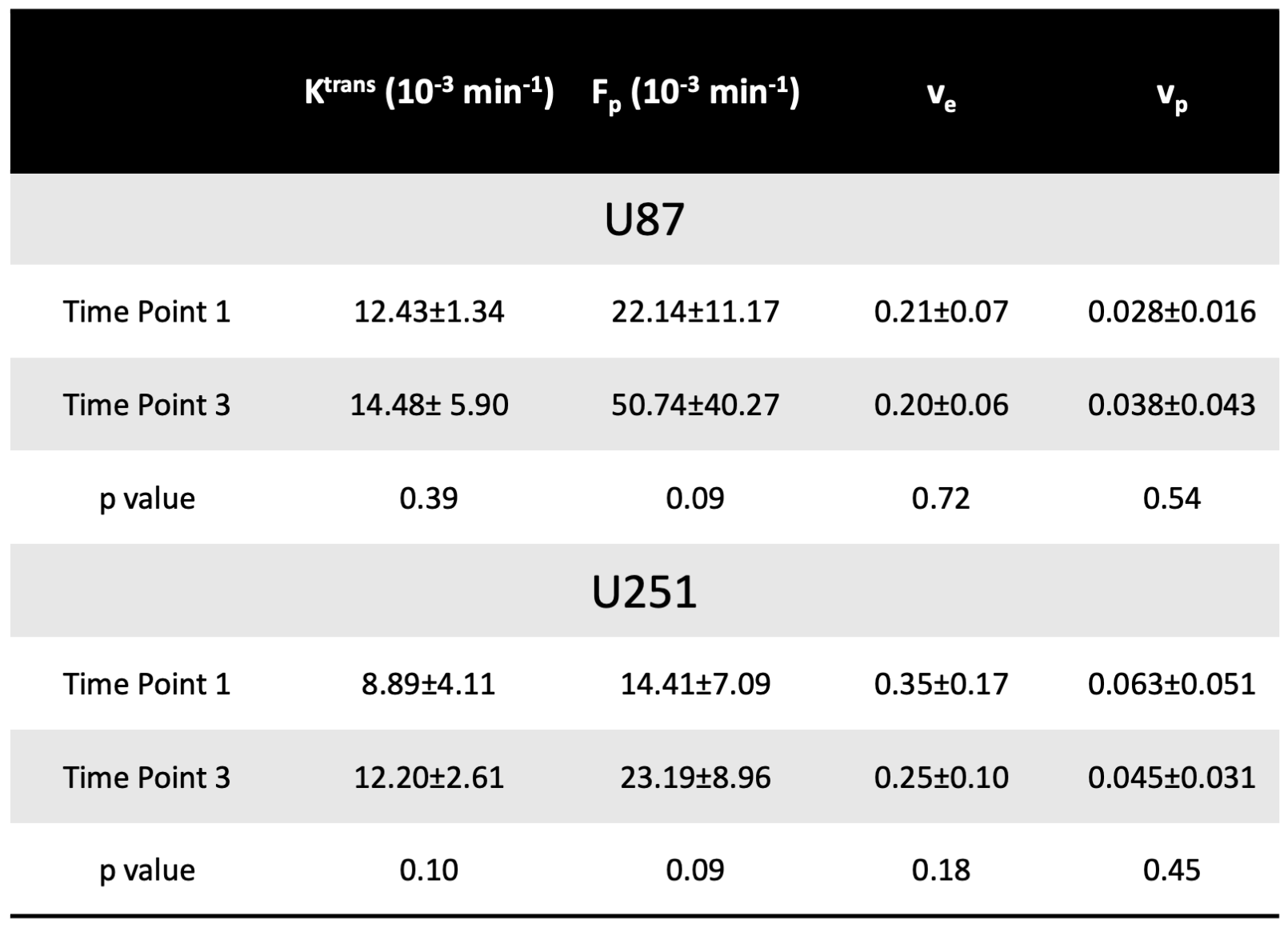

Supplement: Supplementary Table 2 — Quantitative values and statistical comparisons for Ktrans, Fp, ve, and vp vascular parameter values from DCE-MRI for U87 and U251 tumors between the initial and final time point. Parameter values were averaged across all 3 slices from all tumors and are reported as average ± standard deviation. Two tailed t tests are used to compare changes in the parameter values with tumor progression. [file Image_2.tiff]

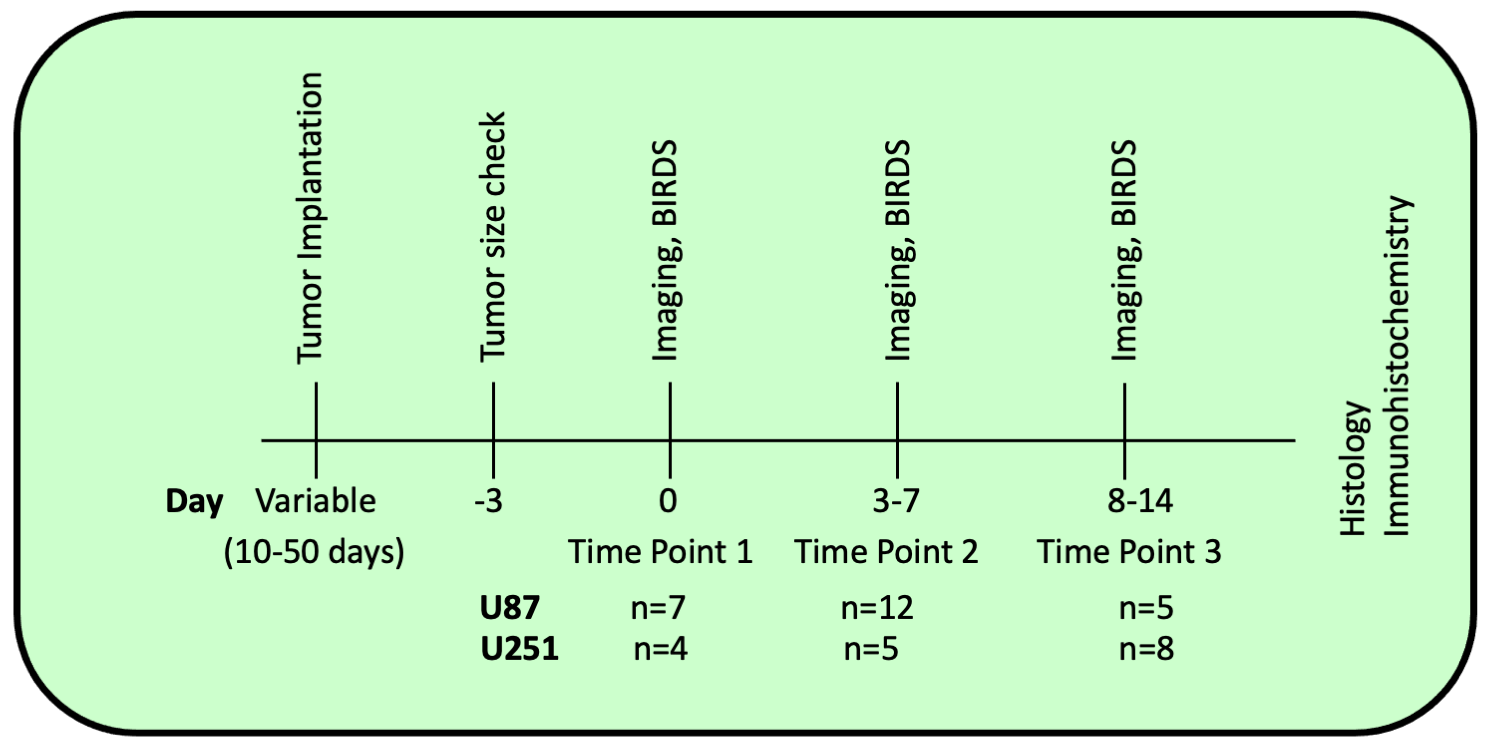

Supplement: Supplementary Figure 1 — Experimental design. Animals were inoculated with either U87 or U251 cells and tumors were allowed to grow. Starting 10 days after inoculation, the animals were scanned to identify the presence of tumor. These scans were repeated every 4-7 days until tumors were identified. After tumor identification, approximately 1-3 days later the first longitudinal scan was performed (Day 0, timepoint 1). These scans were then repeated on day 3-7 and day 8-14. Only data sets in which all imaging modalities were completed (ADC, DCE, BIRDS) were included in the final analysis. [file Image_3.tiff]

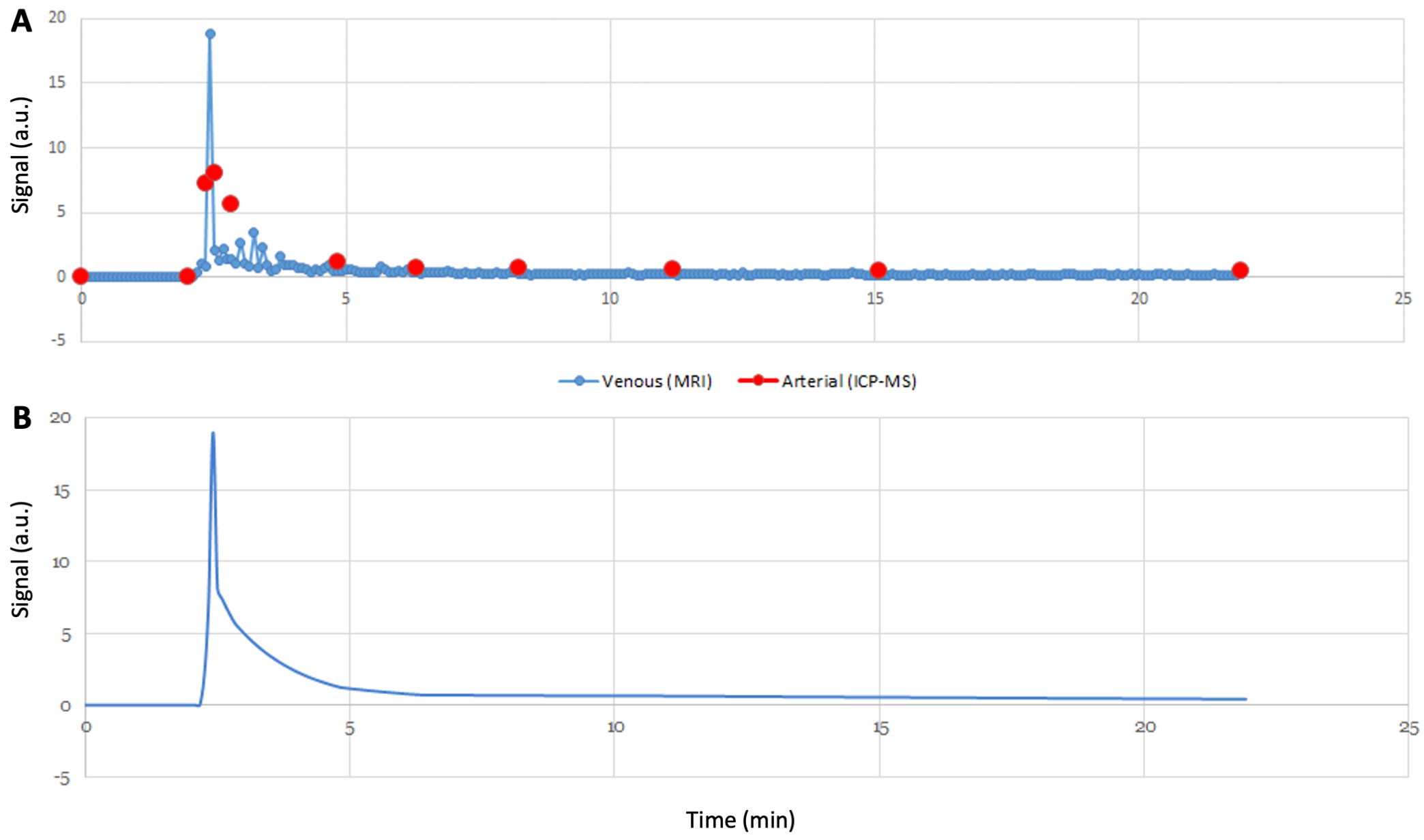

Supplement: Supplementary Figure 2 — AIF used for DCE analysis. The AIF was based on normalized blood contrast agent concentrations obtained from manual arterial blood sampling during gadobutrol infusion in a representative animal (red dots). This was compared with signal intensity measurements taken from the sagittal sinus of the same animal (blue dots). The AIF was fit to a biexponential function (B) which was then utilized for all DCE analysis. [file Image_4.tiff]

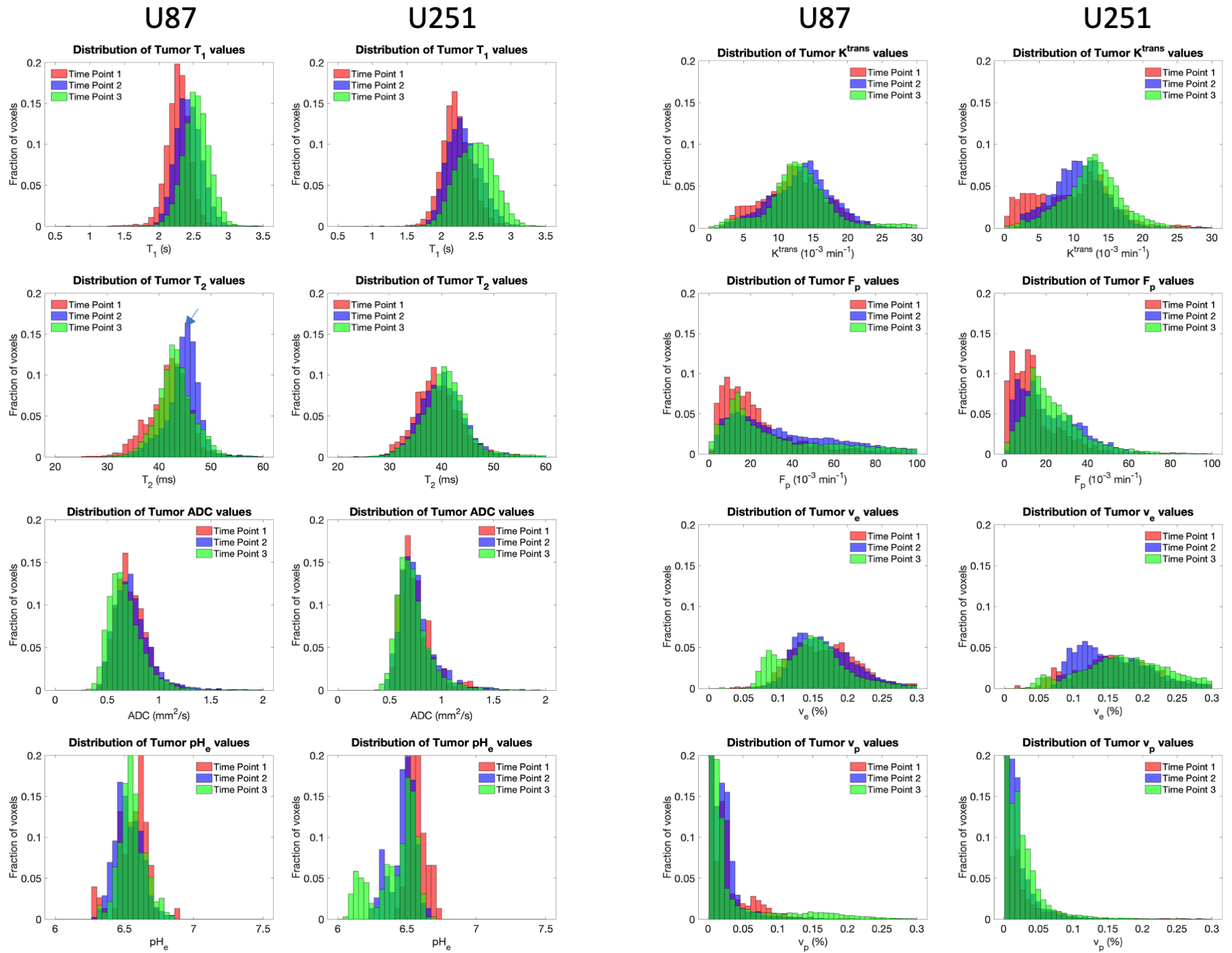

Supplement: Supplementary Figure 3 — Histogram analysis for all parameters measured in U87 and U251 tumors. All tumor voxels are grouped based on the three timepoints and shown as a distribution of parameter values with red being the first timepoint (small tumors), blue the second timepoint, and green the third timepoint (large tumors). [file Image_5.tiff]

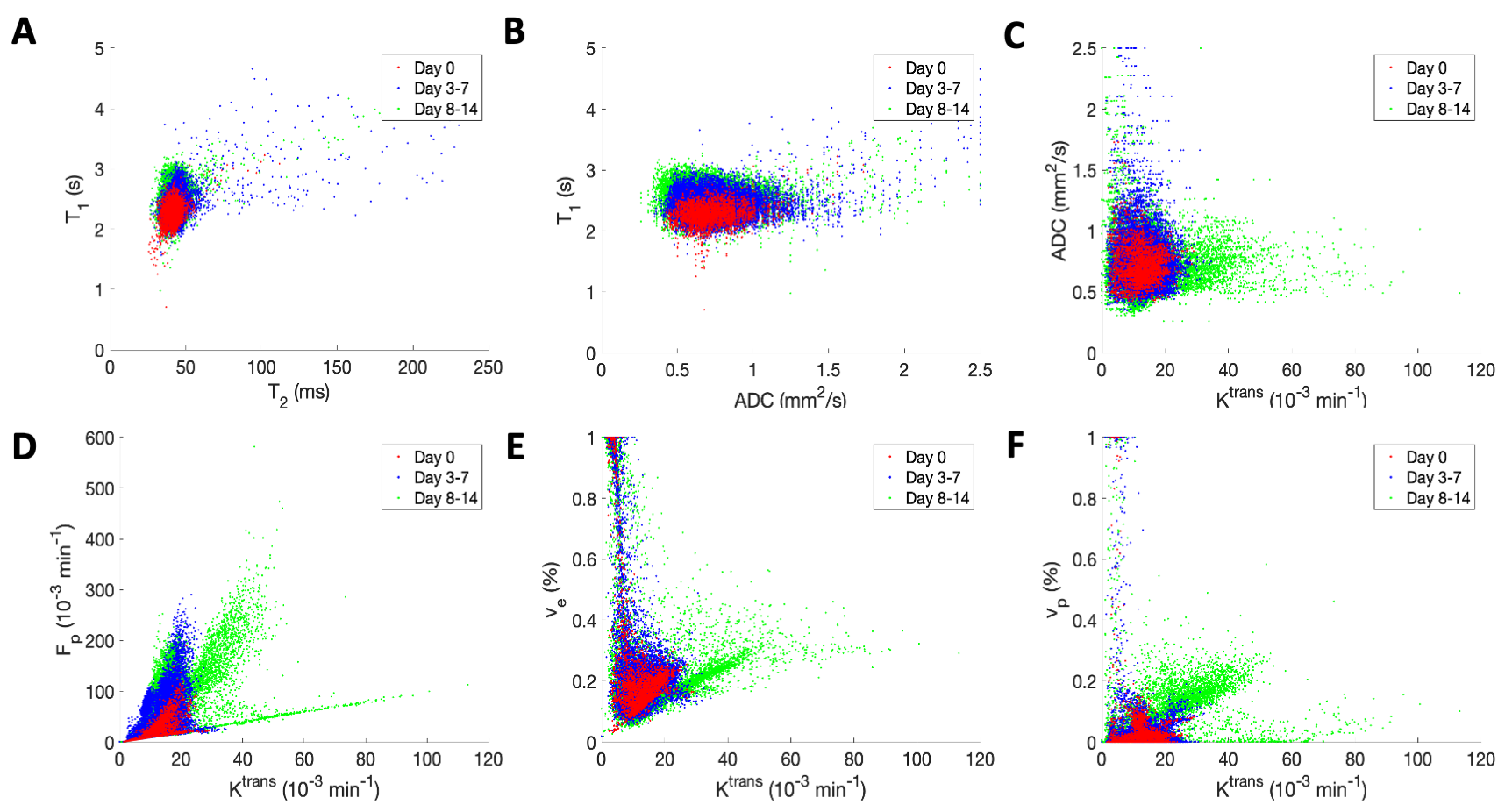

Supplement: Supplementary Figure 4 — MRSI with BIRDS. Voxel-level spectra of TmDOTP5- demonstrating three sharp peaks corresponding to three distinct proton resonances in U87 (A) and U251 (B) tumors. The data show that the agent is primarily located within the tumor with decreasing concentrations in the peritumoral regions, especially in U251 tumors. There is minimal agent accumulation in the normal-appearing brain. [file Image_7.tiff]

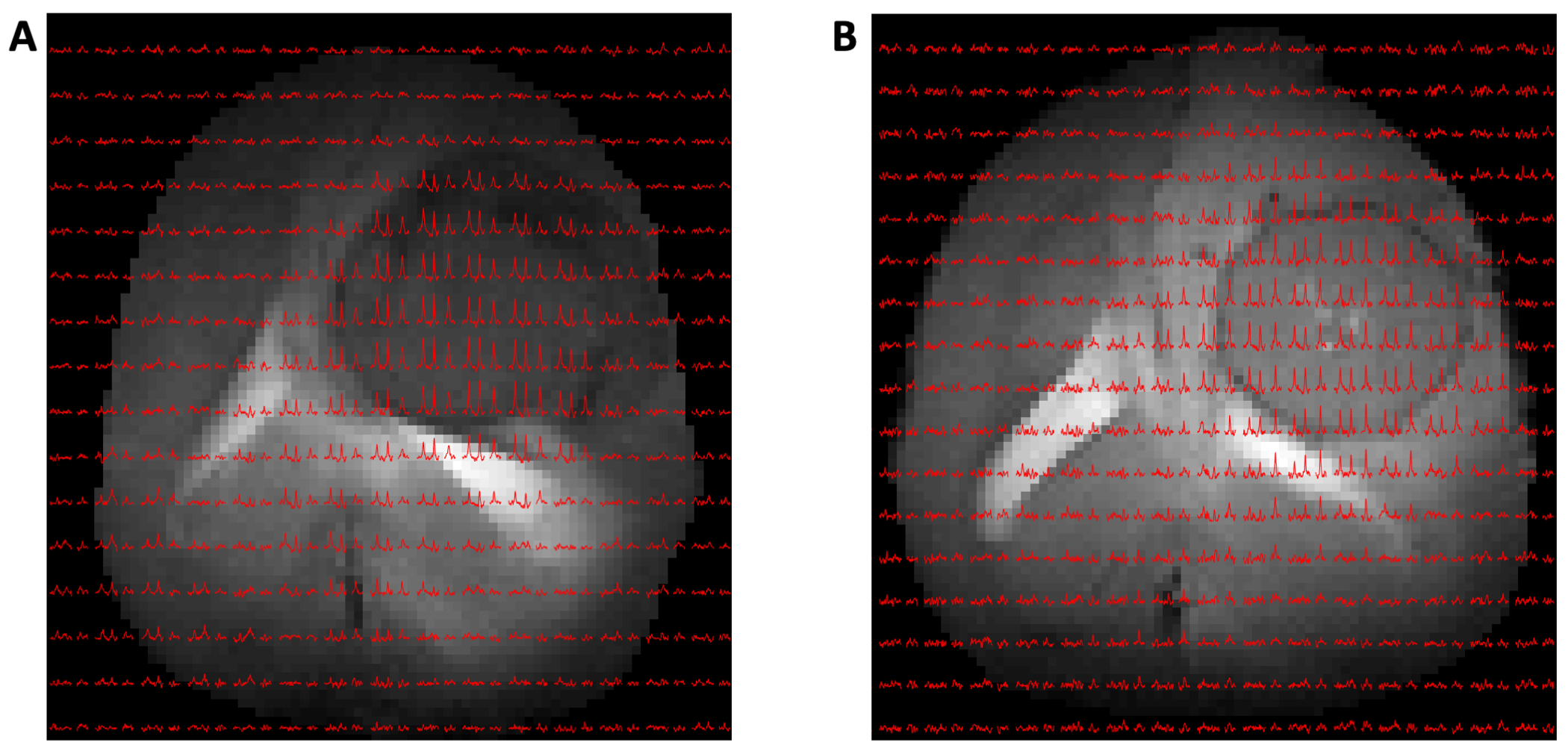

Supplement: Supplementary Figure 5 — Voxel-level analysis for U87 tumors. Measurements for individual voxels could be compared across multiple different parameters. The fraction of voxels with each parameter value were included from all tumors at each timepoint. These relationships demonstrate similar findings to the histogram analysis with increasing distribution of parameter values at later imaging time points (larger tumor volumes). The pHe measurements are not included here because although they were acquired from the same animal, they were acquired with slightly different positioning and at larger voxel sizes, making direct voxel-level correlations with the other parameters challenging. [file Image_6.tiff]
